# Regioisomers Salviprolin A and B, Unprecedented Rosmarinic Acid Conjugated Dinorditerpenoids from *Salvia przewalskii* Maxim

**DOI:** 10.3390/molecules26226955

**Published:** 2021-11-18

**Authors:** Xiangdong Su, Yichuang Wu, Meifang Wu, Jielang Lu, Shujie Jia, Xin He, Shuna Liu, Yuyang Zhou, Hui Xing, Yongbo Xue

**Affiliations:** School of Pharmaceutical Sciences (Shenzhen), Sun Yat-sen University, Shenzhen 518107, China; suxd7@mail.sysu.edu.cn (X.S.); wuych39@mail2.sysu.edu.cn (Y.W.); wumf8@mail2.sysu.edu.cn (M.W.); lujlang@mail2.sysu.edu.cn (J.L.); jiashj@mail2.sysu.edu.cn (S.J.); hexin63@mail2.sysu.edu.cn (X.H.); liushn26@mail2.sysu.edu.cn (S.L.); zhouyy97@mail2.sysu.edu.cn (Y.Z.); xingh5@mail.sysu.edu.cn (H.X.)

**Keywords:** *Salvia przewalskii* Maxim, Lamiaceae, dinorditerpenoids, rosmarinic acid, 1,4-benzodioxane

## Abstract

*Salvia przewalskii* Maxim is a perennial plant from the genus *Salvia* (family Lamiaceae). The roots of *S. przewalskii* were long used as a traditional herb to treat blood circulation related illnesses in China. As part of our continuing interest in polycyclic natural products from medicinal plants, two unprecedented adducts comprised of a *dinor*-diterpenoid and a 9′-*nor*-rosmarinic acid derivative, linked by a 1,4-benzodioxane motif (**1** and **2**), were isolated from the roots of *S. przewalskii*. Their structures were established by extensive spectroscopic approaches including 1D, 2D NMR, and HRFABMS. Their cytotoxic activities against five human tumor cell lines were evaluated.

## 1. Introduction

*Salvia przewalskii* Maxim is a perennial flowering herb which belongs to the genus *Salvia* (family Lamiaceae) [1]. This species is mainly distributed in regions of the Tibetan plateau, such as western parts of the Gansu and Sichuan provinces, as well as northwestern regions of the Yunnan province in China [2]. The roots of *S. przewalskii* were traditionally used as a folk medicine to achieve the therapeutic effects of enhancing blood circulation, remediating blood stasis, regulating menstruation, and relieving pain [3]. According to the Chinese Materia Medica, the roots of *S. przewalskii* were prescribed as a surrogate for Danshen (roots of *S. miltiorrhiza*), a well-known traditional Chinese medicine (TCM) used for the treatment of various cardiovascular diseases [4]. The secondary metabolites from *S. przewalskii* therefore attracted great interest towards their phytochemical investigation in recent decades. Intriguingly, abietane-type diterpenoids and phenolic acid derivatives are two major chemical constituents of the roots of *S. przewalskii*, which also appears in the phytological related *S. miltiorrhiza* [5,6,7,8,9,10]. Pharmacological studies revealed the beneficial effects of both the crude extracts and chemical constituents of *S. przewalskii*, including heart-protective, antioxidative, anti-inflammatory, antitussive, and antibacterial properties [4,11,12,13,14]. This research not only provided convincing evidence to support the traditional therapeutic effects of this species, but has also provided a comprehensive perspective of further potential medicinal applications.

1,4-Benzodioxane is a bicyclic scaffold which consists of a benzene-fused 1,4-dioxane. The versatile structural properties and potential therapeutic effects of this privileged heterocyclic scaffold have resulted in its incorporation in a number of drug design campaigns [15]. Natural products possessing this skeleton were discovered in several plant sources, for instance isoamericanoic acid B from *Acer tegmentosum*, and princepin from *Vitex glabrata* [16,17]. Studies illustrated that the biosynthetic origin of these bicyclic dimers can be traced back to the oxidative dimerization of a number of small molecules, including phenylpropanoids, flavonoids, coumarins, and stilbenoids [18]. Moreover, naturally occurring chiral benzodioxanes demonstrated a range of therapeutic effects, including antiestrogenic, antimalarial, and anti-HCV activities [15].

In our continuing efforts to search for structurally unique and bioactive polycyclic natural products from medicinal plants [19,20,21,22,23], two undescribed phenylpropanoid-diterpenoid adducts (**1** and **2**) possessing a 1,4-benzodioxane scaffold, were encountered from the roots of *S. przewalskii*. Herein, we report the isolation, structural elucidation and evaluation of the cytotoxic effects of these compounds.

## 2. Results

**Figure 1:**
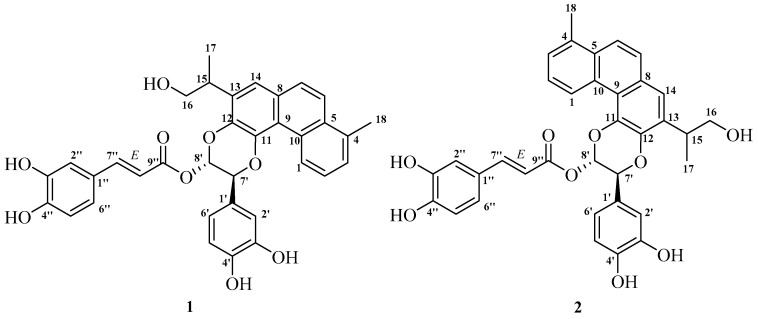
Structures of new compounds **1** and **2**.

A 70% aqueous acetone extract of the air-dried and powdered roots of *S. przewalskii* Maxim was partitioned between H_2_O and CHCl_3_. The CHCl_3_ phase was subjected to repeated column chromatography, and then further purified by semipreparative HPLC to obtain compounds **1** and **2** (Figure 1). Their full structural elucidation was achieved after extensive spectroscopic analyses, including 1D and 2D NMR, and HRFABMS.

Salviprolin A (**1**) was obtained as a dark brown solid. Its molecular formula was determined to be C_35_H_30_O_9_ based on the deprotonated ion peak [M − H]^−^ at *m/z* 593.1817 (calcd for C_35_H_30_O_9_ 593.1812) in the HRFABMS (negative-ion mode) spectrum, indicating 21 degrees of unsaturation (Figure S1). The IR spectrum showed absorption bands characteristic of hydroxyl groups (3423 cm^−1^), a carbonyl group (1703 cm^−1^), aromatic rings (1601, 1521, and 1408 cm^−1^) and ether groups (1284, 1252, and 1112 cm^−1^) (Figure S2).

The ^13^C NMR and DEPT spectra of **1** (Table 1, Figure S5) showed 35 carbon signals, including one ester carbonyl (*δ*_C_ 165.9), 14 quaternary C-atoms (eight aromatic carbons at *δ*_C_ 134.6, 132.2, 129.3, 120.3, 130.6, 134.2, 128.5, and 127.1, and six oxygenated aromatic carbons at *δ*_C_ 141.3, 138.3, 146.2, 146.3, 146.5, and 149.4), 17 methine groups (12 aromatic methines at *δ*_C_ 127.4, 126,4, 128.1, 122.4, 127.9, 120.9, 114.8, 116.2, 119.5, 115.5, 116.4, and 123.0, two olefinic methines at *δ*_C_ 148.0 and 114.0 and three aliphatic methines including two oxygenated carbons at *δ*_C_ 36.6, 76.0, and 90.4), one oxygenated methylene (*δ*_C_ 67.0) and two methyl groups (*δ*_C_ 17.1 and 20.5).

**Figure 2:**
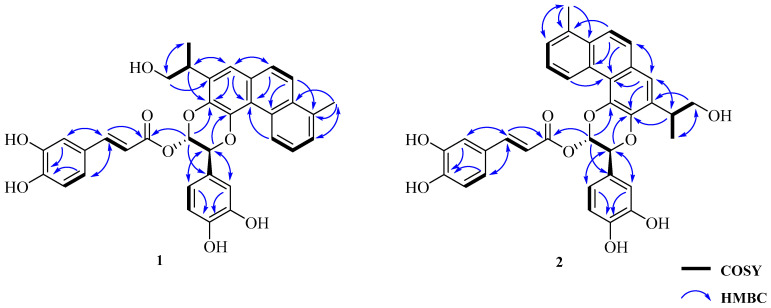
Key COSY and key HMBC correlations of compounds **1** and **2**.

The ^1^H NMR spectrum of **1** displayed a set of signals consistent with a 1,2,3-trisubstituted phenyl core [*δ*_H_ 9.61 (1H, m, H-1), 7.43 (2H, overlapping, H-2 and H-3)], a 1,2,3,4-tetrasubstituted phenyl ring [*δ*_H_ 7.90 (1H, d, *J* = 9.2 Hz, H-6) and 7.77 (1H, d, *J* = 9.2 Hz, H-7)], and a singlet aromatic proton [*δ*_H_ 7.51 (1H, s, H-14)] (Table 1, Figure S4). In the ^1^H-^1^H COSY spectrum (Figure 2 and Figure S6), a spin-coupling system connecting an oxygenated methylene [H-16 (*δ*_H_ 3.65 and 3.85)] to a methine [H-15 (*δ*_H_ 3.42)], and continuing to a methyl group [H-17 (*δ*_H_ 1.35)], established the 1-isopropanol segment of CH_2_(16)-CH(15)-CH_3_(17). Since the 1D-NMR data of **1** were indicative of a highly oxygenated polycyclic structure unlike all previously known diterpenoids isolated from the *salvia* genus, a comprehensive analysis of its 2D-NMR spectroscopic data was conducted.

The HMBC correlations from H-15 (*δ*_H_ 3.42) to C-12 (*δ*_C_ 138.3)/C-14 (*δ*_C_ 120.9), from H-14 to C-15 (*δ*_C_ 36.6), together with the HMBC correlations from H-16 (*δ*_H_ 3.65 and 3.85)/H-17 (*δ*_H_ 1.35) to C-13 (*δ*_C_ 134.2) confirmed the C-C linkage of C-15 to C-13 (Figure 2 and Figure S7). Moreover, the HMBC correlations from H-18 (*δ*_H_ 2.73) to C-3 (*δ*_C_ 128.1)/C-5 (*δ*_C_ 132.2) were employed to locate a methyl group at C-4 (Figure 2 and Figure S7). This led us to deduce the presence of a dinorditerpenoid residue, a structural motif which was observed in *R*-(+)-salmiltiorin E [24].

**Figure 3:**
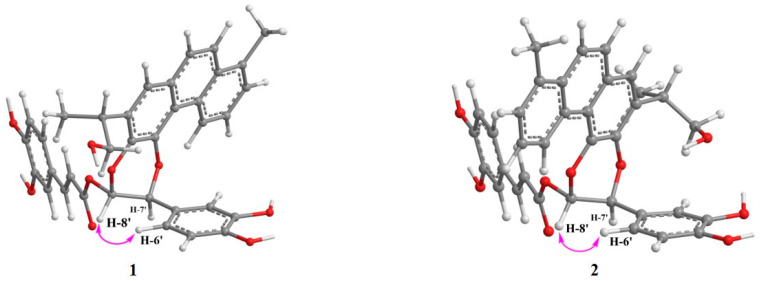
Energy-minimized structures of compounds **1** and **2** with Key ROE correlations.

The additional ^1^H NMR data demonstrated a 1,3,4-trisubstrituted phenyl ring [*δ*_H_ 7.12 (1H, d, *J* = 1.9 Hz, H-2′), 6.84 (1H, d, *J* = 8.0 Hz, H-5′), 6.99 (1H, dd, *J* = 1.9, 8.0 Hz, H-6′)] skeleton typical of a caffeoyl moiety [*δ*_H_ 7.16 (1H, d, *J* = 1.5 Hz, H-2″), 6.84 (1H, d, *J* = 8.0 Hz, H-5″), 7.04 (1H, dd, *J* = 1.5, 8.0 Hz, H-6″), 7.64 (1H, d, *J* = 15.8 Hz, H-7″), 6.29 (1H, d, *J* = 15.8 Hz, H-8″)] (Table 1, Figure S4) [25]. In addition, the *trans*-coupled H-7″ and H-8″ were elucidated on the basis of their relatively large coupling constants (*J* = 15.8 Hz). The HMBC spectrum of **1** revealed HMBC correlations from H-7′ (*δ*_H_ 5.52) to C-11 (*δ*_C_ 141.3), and from H-8′ (*δ*_H_ 6.77) to C-12 (*δ*_C_ 138.3). Taken together with the ^1^H-^1^H COSY correlation between H-7′ and H-8′, this established the coexistence of two ether linkages of CH(7′)-*O*-C(11) and CH(8′)-*O*-C(12), ultimately suggesting the diterpenoid was fused at C-11 and C-12 by the aforementioned phenyl-1,4-benzodioxane moiety (Figure 2 and Figure S7). The subsequent key HMBC correlations from H-7′ (*δ*_H_ 5.52) to C-2′ (*δ*_C_ 114.8), C-6′ (*δ*_C_ 119.5) and the HMBC interactions from H-2′ (*δ*_H_ 7.12)/H-6′ (*δ*_H_ 6.99) to C-7′ (*δ*_C_ 76.0) indicated the 1,3,4-trisubstrituted phenyl ring was attached to the phenyl-1,4-benzodioxane moiety at C-7′ (Figure 2 and Figure S7). Likewise, key HMBC correlations from H-8′ (*δ*_H_ 6.77) to C-9″ (*δ*_C_ 165.9) and C-1′ (*δ*_C_ 128.5) supported the conclusion that the *trans*-caffeoyl residue was linked to the phenyl-1,4-benzodioxane moiety at C-8′ by an ester group. Hence, the planar structure of **1** was determined as shown (Figure 2). Furthermore, the coupling constant between H-7′ and H-8′ (*J* = 3.8 Hz) was very close to that of H-7″ and H-8″ (*J* = 3.3 Hz) (i.e., similar to that of an oxidized isoamericanol A), and a ROE correlation between H-8′ and H-6′ as shown (Figure 3 and Figure S9) suggesting the two protons at C-7′ and C-8′ to be *trans*-oriented. It was based on these that the 7′R*, 8′S*-configuration of **1** was provisionally assigned [26,27,28]. Consequently, the three-dimensional structure of **1** was determined as shown (Figure 1), and salviprolin A was assigned.

Compound **2** was obtained as a dark brown solid, which had the same molecular formula as **1**, C_35_H_30_O_9_, as deduced from a quasi-molecular ion peak in its HR-FAB-MS spectrum (Figure S12). The ^1^H and ^13^C NMR spectroscopic data of **2** were almost identical to those of **1** (Table 1, Figures S13 and S14), which suggested a large degree of structural similarity between the two compounds. Key HMBC correlations observed in the HMBC spectrum from H-7′ (*δ*_H_ 5.36) to C-12 (*δ*_C_ 140.4) and H-8′ (*δ*_H_ 6.74) to C-11 (*δ*_C_ 139.1), indicated the presence of an ether-linkage from C-12 to C-7′ and from C-11 to C-8′, respectively, instead of CH(7′)-*O*-C(11) and CH(8′)-*O*-C(12) as displayed in **1** (Figure S16). Thus, compound **2** was determined to be a regioisomer of **1** at the C-7′ and C-8′ positions. The ROE correlation between H-8′ and H-6′ suggested H-7′ and H-8′ to be *trans*-oriented as shown (Figure 3 and Figure S18). Moreover, the coupling constant between H-7′ and H-8′ of **2** was almost identical to those of **1**, thereby indicating a 7′R*, 8′S*-configuration of **2**. The structure of compound **2** was therefore determined as shown (Figure 1) and named salviprolin B.

Compounds **1** and **2** were evaluated for in vitro cytotoxicity against the human tumor cell lines HL-60, SMMC-7721, A-549, MCF-7, and SW480 cell lines using the MTT method as previously reported [29]. *cis*-Platin (Sigma) was used as the positive control. Unfortunately, both compounds were found to be inactive with IC_50_ values of >40 μM (Table 2).

## 3. Discussion

*Origanum dictamnus* L. (family Lamiaceae) was previously reported to produce a series of benzodioxane-containing metabolites, for example, salvianolic acid P [30]. Interestingly, our current work on *S. przewalskii* from the Lamiaceae family also resulted in characterization of two unique *nor*-phenylpropanoid and *dinor*-diterpenoid adducts (**1** and **2**) containing a 1,4-benzodioxane nucleus. The above findings may suggest a similar biosynthetic pathway between these two species of the Lamiaceae family. To the best of our knowledge, this is the first report of natural chiral 1,4-benzodioxane adducts containing a *nor*-rosmarinic acid derivative and a *dinor*-diterpenoid. Derivatives of rosmarinic acid such as salvianolic acid, and dinorditerpenoids astanshinones are widely distributed in species from the Lamiaceae family, especially in the genus *Salvia* [31,32]. As with many constituents of *S. przewalskii* and *S. miltiorrhiza*, rosmarinic acid derivatives and dinorditerpenoids also exhibit a wide range of therapeutic benefits, including anti-tumor, antioxidant, anti-inflammatory, and antibacterial properties [31,32,33]. Unfortunately, due to poor isolation yields, the biological evaluation of compounds **1** and **2** in this study was limited to a preliminary investigation into their cytotoxic activity. The privileged nature of their unique 1,4-benzodioxane motifs that feature both a hydrophilic phenolic moiety and a lipophilic diterpenoid, however, hold great potential for promising bioactivity in other therapeutic contexts. It is our hope that either improvement and scale-up of the isolation methods or a total synthesis approach will be able to provide quantities of **1** and **2** enough to facilitate their further pharmacological evaluation.

## 4. Experimental

### 4.1. General Experimental Procedures

Optical rotations were measured with a Horiba SEPA-300 polarimeter. UV spectra were recorded using a Shimadzu UV-2401A spectrophotometer equipped with a DAD and a 1 cm pathlength cell. Methanolic samples were scanned from 190 to 400 nm in 1 nm steps. IR spectra were obtained using a Tenor 27 spectrophotometer with KBr pellets. 1D and 2D NMR spectra were acquired on a Bruker DRX-500 spectrometer with TMS as internal standard. Chemical shifts (δ) were expressed in ppm with reference to the solvent signals. Mass spectra were recorded on a VG Auto Spec-3000 instrument or an API QSTAR Pulsar 1 spectrometer. Semipreparative HPLC was performed on an Agilent 1100 apparatus equipped with a UV detector and a Zorbax SB-C-18 (Agilent, 9.4 mm × 25 cm) column. Column chromatography was performed using silica gel (200–300 mesh and H, Qingdao Marine Chemical Co. Ltd., Qingdao, China), RP-18 gel (40–63 μm, Merck, Darmstadt, Germany), and MCI gel (75–150 μm; Mitsubishi Chemical Corporation, Japan). Fractions were monitored by TLC (GF254, Qingdao Marine Chemical Co. Ltd., Qingdao, China), and spots were visualized by heating silica gel plates sprayed with 10% H_2_SO_4_ in EtOH. All solvents were distilled prior to use.

### 4.2. Plant Materials

The air-dried and powdered roots of *S. przewalskii* Maxim (7.0 kg) were collected at Zhongdian County, Yunnan province, People’s Republic of China, on August 2005, and the plant was identified by Prof. Xiao Cheng at Kunming Institute of Botany, Chinese Academy of Sciences. A specimen (No. 20050623 L2) was deposited in Kunming Institute of Botany, Chinese Academy of Sciences.

### 4.3. Extraction and Isolation

The air-dried and powdered roots of *S. przewalskii* Maxim (7.0 kg) were extracted with 70% aqueous acetone (24 h × 3 times) at room temperature and concentrated in vacuo to give a crude extract (1.3 kg). The extract was suspended in H_2_O, and then extracted with CHCl_3_. The CHCl_3_-soluble extract (220.0 g) was chromatographed over a silica gel chromatography column (CC) (petroleum ether/acetone from 1:0 to 0:1) to give fractions SI–VII. Fr. SI was subjected to silica gel CC (petroleum ether/EtOAc from 20:1 to 0:1) to afford six subfractions (SI1–SI6). Fr. SI3 (647.0 mg) was subjected to Sephadex LH-20 gel CC (CHCl_3_/MeOH, 1:1) to give four subfractions (SI3A–SI3D). Fr. SI3C was purified by silica gel CC (petroleum ether/CHCl_3_/EtOAc, 70:28:2) to yield seven subfractions (SI3C1–SI3C7). Fr. SIII (6.3 g) was chromatographed over silica gel CC (petroleum ether/EtOAc, 16:1–2:3) to afford eight subfractions (SIII1–III8). Fr. SIII5 (648.0 mg) was purified by semipreparative HPLC (MeOH/H_2_O, 85:15) to yield compounds **1** (3.2 mg) and **2** (4.0 mg).

### 4.4. Salviprolin A (***1***)

Dark brown solid; ${\left[\alpha \right]}_{D}^{26.2}$: −69.8° (*c* 1.21, MeOH); UV (MeOH) *λ*_max_ (log *ε*) 363 (2.2), 281 (2.4), 258 (2.7), 212 (2.8); IR (KBr) *ν*_max_ 3423, 2956, 1703, 1601, 1521, 1408, 1284, 1252, 1112, 804, 762 cm^−1^; ^1^H NMR and ^13^C NMR see Table 1; negative FABMS *m/z* 593 ([M − H]^−^; negative HRFABMS *m/z* 593.1817 (calcd. for C_35_H_29_O_9_, 593.1812).

### 4.5. Salviprolin B (***2***)

Dark brown solid; ${\left[\alpha \right]}_{D}^{26.2}$: −204.2° (*c* 0.48, MeOH); UV (MeOH) *λ*_max_ (log *ε*) 364 (2.6), 281 (2.8), 258 (3.1), 209 (3.1); IR (KBr) *ν*_max_ 3431, 2959, 1703, 1608, 1523, 1383, 1285, 1200, 980, 763 cm^−1^; ^1^H NMR and ^13^C NMR see Table 1; negative FABMS *m/z* 593 ([M − H]^−^; negative HRFABMS *m/z* 593.1804 (calcd. for C_35_H_29_O_9_, 593.1812).

### 4.6. Cytotoxic Assays

Colorimetric assays were performed to evaluate compound activity [29]. The following human tumor cell lines were used: the A549 lung cancer cell line, the HL-60 human myeloid leukemia cell line, the MCF-7 breast cancer cell line, the SMMC-7721 human hepatocarcinoma cell line, and the SW-480 human pancreatic carcinoma. All cells were cultured in RPMI-1640 or DMEM medium (Hyclone, Logan, UT, USA), supplemented with 10% fetal bovine serum (Hyclone) at 37 °C in a humidified atmosphere with 5% CO_2_. Cell viability was assessed by conducting colorimetric measurements of the amount of insoluble formazan formed in living cells based on the reduction of 3-(4,5-dimethylthiazol-2-yl)-2,5-diphenyltetrazolium bromide (MTT) (Sigma, St. Louis, MO, USA). Briefly, 100 μL adherent cells were seeded into each well of a 96-well cell culture plate and allowed to adhere for 12 h before compound addition, while suspended cells were seeded just before compound addition, both with initial density of 1 × 105 cells/mL in 100 μL of medium. Each tumor cell line was exposed to the test compound at various concentrations in triplicate for 48 h, with *cis*-Platin (Sigma) as positive control. After incubation, MTT (100 μg) was added to each well, and the incubation was continued for 4 h at 37 °C. The cells were lysed with 100 μL of 20% SDS-50% DMF after removal of 100 μL of medium. The optical density of the lysate was measured at 595 nm in a 96-well microtiter plate reader (Bio-Rad 680). The IC_50_ value of each compound was calculated by Reed and Muench’s method.

## Figures and Tables

**Table 1:** ^1^H (400 MHz) and ^13^C (100 MHz) NMR data, and HMBC correlations for **1**^a^ and **2** ^a^.

No	1			2		
	*δ_C_*	*δ_H_* (Mult, *J*, Hz)	HMBC	*δ_C_*	*δ_H_* (Mult, *J*, Hz)	HMBC
1	127.4	9.61 m	3, 5, 9	127.3	9.44 m	3, 5, 9
2	126.4	7.43 ^c^	4, 10	126.3	7.40 ^c^	4, 10
3	128.1	7.43 ^c^	1, 4, 5, 18	128.1	7.40 ^c^	1, 4, 5, 18
4	134.6	-		134.5	-	
5	132.2	-		132.2	-	
6	122.4	7.90 d (9.2)	4, 8, 10	122.1	7.85 d (9.1)	4, 8, 10
7	127.9	7.77 d (9.2)	5, 6, 8, 9, 14	127.7	7.75 d (9.1)	5, 8, 9, 14
8	129.3	-		128.9	-	
9	120.3	-		120.5	-	
10	130.6	-		130.3	-	
11	141.3	-		139.1	-	
12	138.3	-		140.4	-	
13	134.2	-		134.0	-	
14	120.9	7.51 s	7, 9, 12, 15	121.4	7.56 s	7, 9, 12, 15
15	36.6	3.42 m	12, 13, 14, 16, 17	36.5	3.57 m	12, 14, 16, 17
16	67.0	3.65 dd (10.4, 7.2)	13, 15, 17	67.1	3.70 dd (10.4, 7.2)	13, 15, 17
		3.85 dd (10.4, 5.6)			3.92 dd (10.4, 5.6)	
17	17.1	1.35 d (5.6)	13, 15, 16	17.3	1.42 d (6.8)	13, 15, 16
18	20.5	2.73 s	3, 4, 5	20.5	2.69 s	3, 4, 5
1′	128.5	-		128.6	-	
2′	114.8	7.12 d (1.9)	1′, 3′, 4′, 6′, 7′	114.9	7.11 br s	4′, 6′
3′	146.2 ^b^	-		146.1 ^b^	-	
4′	146.3 ^b^	-		146.4 ^b^	-	
5′	116.2 ^b^	6.84 ^c^ d (8.0)	1′, 3′, 4′, 6′	116.1 ^b^	6.85 ^c^	1′, 3′
6′	119.5	6.99 dd (8.0, 1.9)	1′, 2′, 4′, 7′	119.7	6.97 d (7.8)	2′, 4′, 5′, 7′
7′	76.0	5.52 d (3.8)	1′, 2′, 6′, 8′, 11	76.1	5.36 d (4.1)	1′, 2′, 6′, 8′, 12
8′	90.4	6.77 d (3.8)	1′, 7′, 9″, 12	90.8	6.74 d (4.1)	1′, 7′, 9″, 11
1″	127.1	-		127.0	-	
2″	115.5	7.16 d (1.5)	4″, 6″, 7″	115.5	7.17 br s	4″, 6″
3″	146.5	-		146.6 ^b^	-	
4″	149.4	-		149.4	-	
5″	116.4 ^b^	6.84 ^c^ d (8.0)	1″, 3″, 4″, 6″	116.4 ^b^	6.85 ^c^	1″, 3″, 4″, 6″
6″	123.0	7.04 dd (8.0, 1.5)	2″, 4″, 7″	123.0	7.02 d (7.8)	2″, 4″, 5″
7″	148.0	7.64 d (15.8)	1″, 2″, 6″, 9″	148.2	7.64 d (15.8)	1″, 2″, 6″, 9″
8″	114.0	6.29 d (15.8)	1″, 7″, 9″	113.8	6.30 d (15.8)	1″, 9″
9″	165.9	-		165.9	-	

^a^ NMR data were recorded in acetone-*d*_6_; ^b^ assignments may be interchanged; ^c^ overlapped peaks.

**Table 2:** Cytotoxic activity of compounds **1** and **2** ^a^.

Compound	A-549	HL-60	MCF-7	SMMC-7721	SW-480
**1**	>40	>40	>40	>40	>40
**2**	>40	>40	>40	>40	>40
*cis*-platin	15.6	0.9	14.9	13.8	19.1

^a^ Results are expressed as IC_50_ values in μM; data were obtained from triplicate experiments; *cis*-platin was used as a positive control.

## Data Availability

Supporting Information data include HRFABMS, IR, UV and 1D, 2D NMR spectral charts.

## Supplementary Materials

The following are available online. The HR-FAB-MS, IR, UV and 1D, 2D NMR spectra of compounds **1** and **2** are available in supplementary materials. Figure S1: HRFABMS spectrum of **1**, Figure S2: IR spectrum of **1** (KBr), Figure S3: UV spectrum of **1** (MeOH), Figure S4: ^1^H NMR spectrum of **1** in acetone-*d*_6_, Figure S5: ^13^C NMR spectrum of **1** in acetone-*d*_6_, Figure S6: COSY spectrum of **1** in acetone-*d*_6_, Figure S7: HMBC spectrum of **1** in acetone-*d*_6_, Figure S8: HSQC spectrum of **1** in acetone-*d*_6_, Figure S9: ROESY spectrum of **1** in acetone-*d*_6_, Figure S10: UV spectrum of **2** (MeOH), Figure S11: IR spectrum of **2** (KBr), Figure S12: HRFABMS spectrum of **2,** Figure S13: ^1^H NMR spectrum of **2** in acetone-*d*_6_, Figure S14: ^13^C NMR spectrum of **2** in acetone-*d*_6_, Figure S15: HSQC spectrum of **2** in acetone-*d*_6_, Figure S16: HMBC spectrum of **2** in acetone-*d*_6_, Figure S17: COSY spectrum of **2** in acetone*-d*_6_, Figure S18: ROESY spectrum of **2** in acetone-*d*_6_.

## Sample Availability

Samples of the compounds **1** and **2** are available from the authors.
