# Supplementary material for: Regioisomers Salviprolin A and B, Unprecedented Rosmarinic Acid Conjugated Dinorditerpenoids from Salvia przewalskii Maxim
Source: Molecules. 2021 Nov 18;26(22):6955. doi: 10.3390/molecules26226955 (PMC8618536; doi:10.3390/molecules26226955)
Supplement: Supplementary file 1 [file molecules-26-06955-s001.zip › molecules-1441456-SI.pdf]

molecules

MDPI

Article

Regioisomers Salviprolin A and B, Unprecedented Rosmarinic Acid Conjugated Dinorditerpenoids from *Salvia przewalskii* Maxim

Xiangdong Su, Yichuang Wu, Meifang Wu, Jielang Lu, Shujie Jia, Xin He, Shuna Liu, Yuyang Zhou, Hui Xing and Yongbo Xue *

School of Pharmaceutical Sciences (Shenzhen), Sun Yat-sen University, Shenzhen 518107, China; suxd7@mail.sysu.edu.cn (X.S.); wuych39@mail2.sysu.edu.cn (Y.W.); wumf8@mail2.sysu.edu.cn (M.W.); lujlang@mail2.sysu.edu.cn (J.L.); jiashj@mail2.sysu.edu.cn (S.J.); hexin63@mail2.sysu.edu.cn (X.H.); liushn26@mail2.sysu.edu.cn (S.L.); zhouyy97@mail2.sysu.edu.cn (Y.Z.); xingh5@mail.sysu.edu.cn (H.X.)

* Correspondence: xueyb@mail.sysu.edu.cn

Supporting Information

Fig. S1. UV spectrum of 1 (MeOH)……………………………………..…………2

Fig. S2. IR spectrum of 1 (KBr) ……………………………………...……..………2

Fig. S3. HRFABMS spectrum of **1**………………………………….…...…………3

Fig. S4. ¹H NMR spectrum of **1** in acetone-d_6……………………...……...……..……3

Fig. S5. ¹³C NMR spectrum of **1** in acetone-d_6…………………….……...……..…..4

Fig. S6. HSQC spectrum of **1** in acetone-d_6………………………….…......……4

Fig. S7. HMBC spectrum of **1** in acetone-d_6……………………….……...……......……5

Fig. S8 COSY spectrum of **1** in acetone-d_6………………………...………....……5

Fig. S9 ROESY spectrum of **1** in acetone-d_6…..……………………..……...…….........6

Fig. S10. UV spectrum of **2** (MeOH)……………………….…………...………......……6

Fig. S11. IR spectrum of **2** (KBr). ……………………..……………...…….. .....……7

Fig. S12. HRFABMS spectrum of **2**…………………….…………………..... ..……..7

Fig. S13. ¹H NMR spectrum of **2** in acetone-d_6……………………...……............…..8

Fig. S14. ¹³C NMR spectrum of **2** in acetone-d_6……………….…...……........……..8

Fig. S15. HSQC spectrum of **2** in acetone-d_6……………………….…...……..........…...9

Fig. S16. HMBC spectrum of **2** in acetone-d_6……………………...……......……9

Fig. S17. COSY spectrum of **2** in acetone-d_6…………………..……..……......……10

Fig. S18 ROESY spectrum of **2** in acetone-d_6…...……………….……...………...…..10

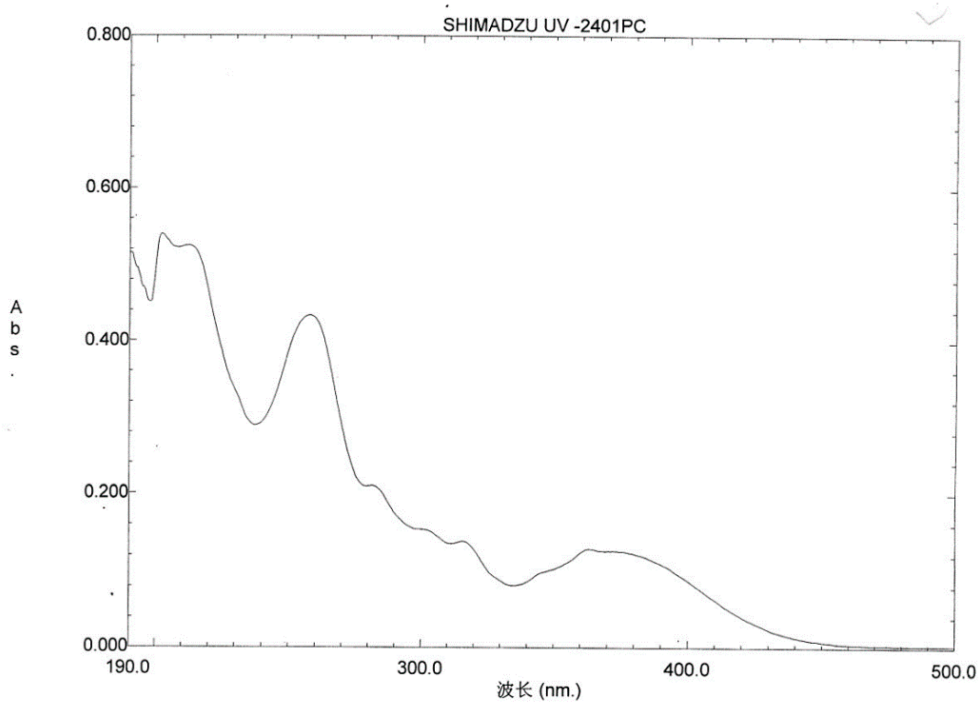

Figure S1. UV spectrum of **1** (MeOH).

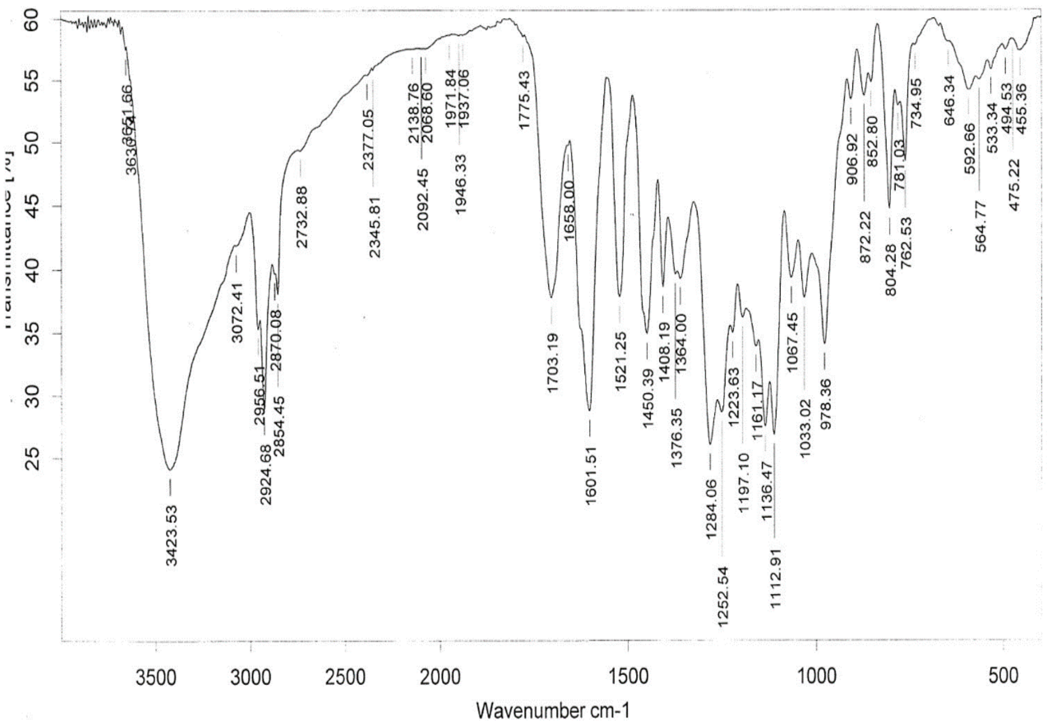

Figure S2. IR spectrum of **1** (KBr).

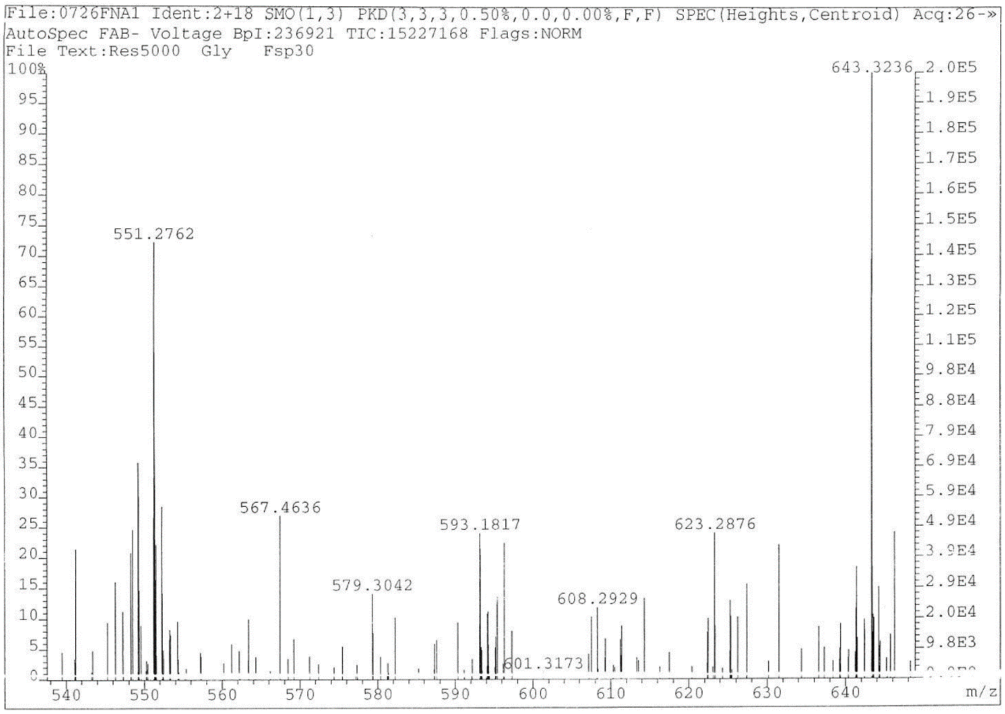

Figure S3. HRFABMS spectrum of **1.**

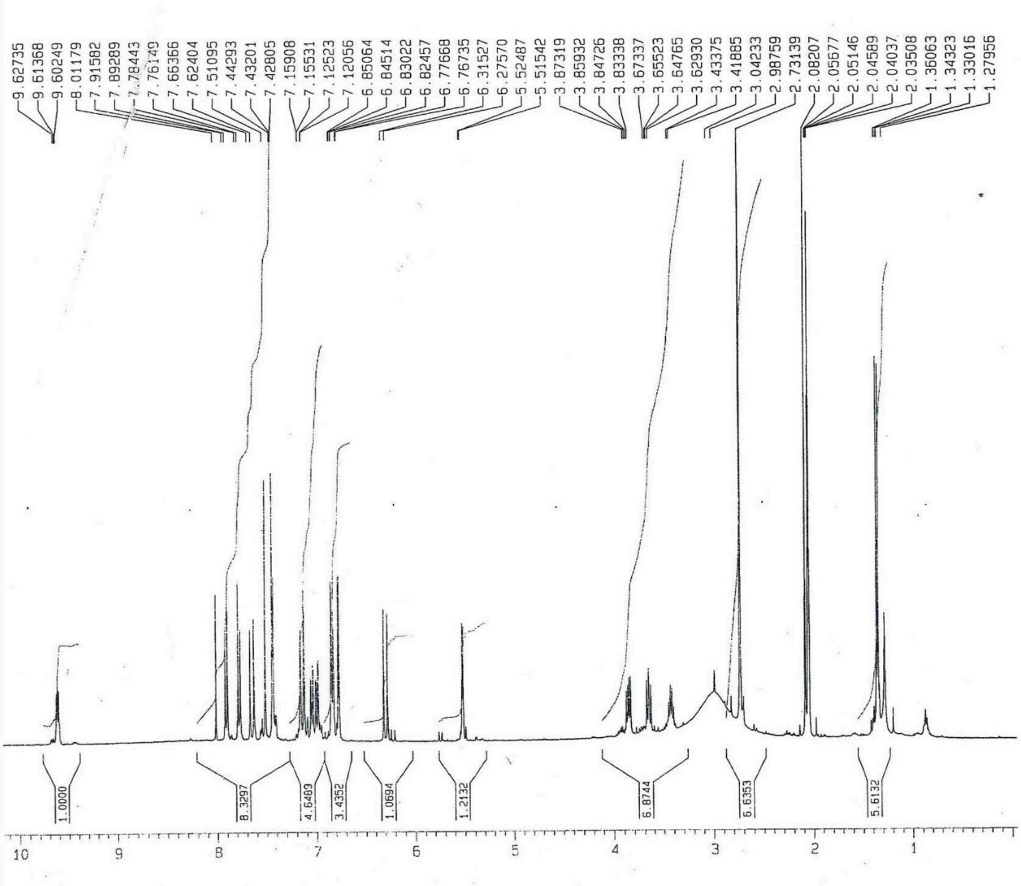

Figure S4. ^{1}H NMR spectrum of **1** in acetone-d_6.

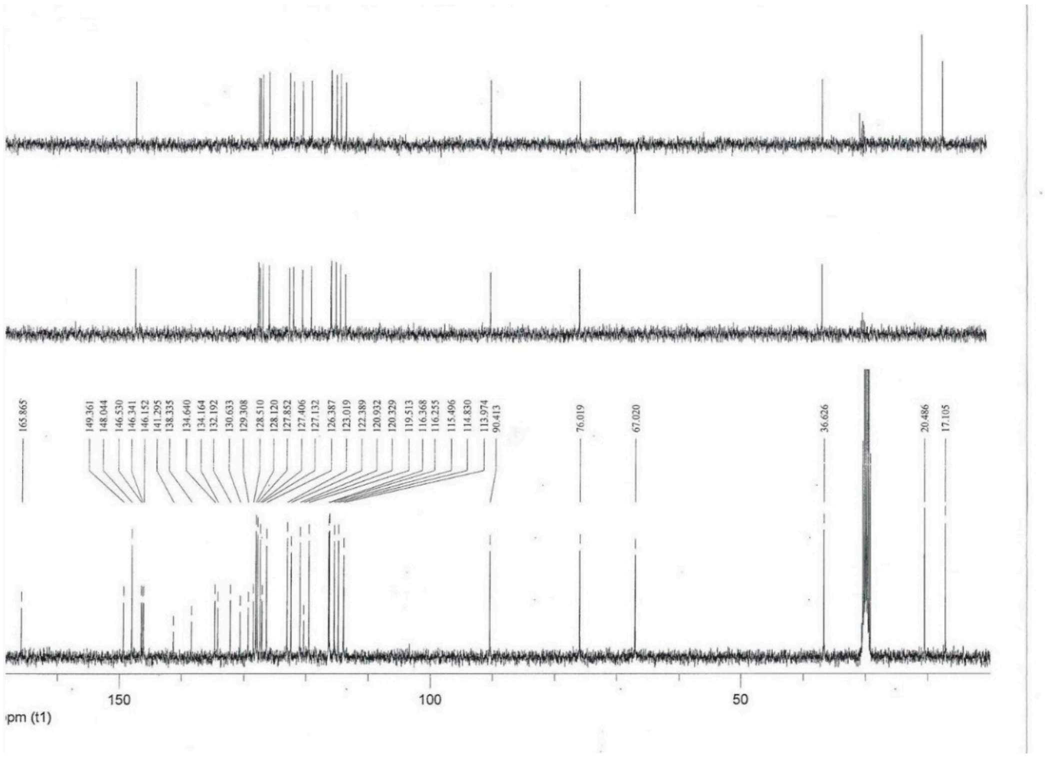

Figure S5. ^{13}C NMR spectrum of **1** in acetone-d_6.

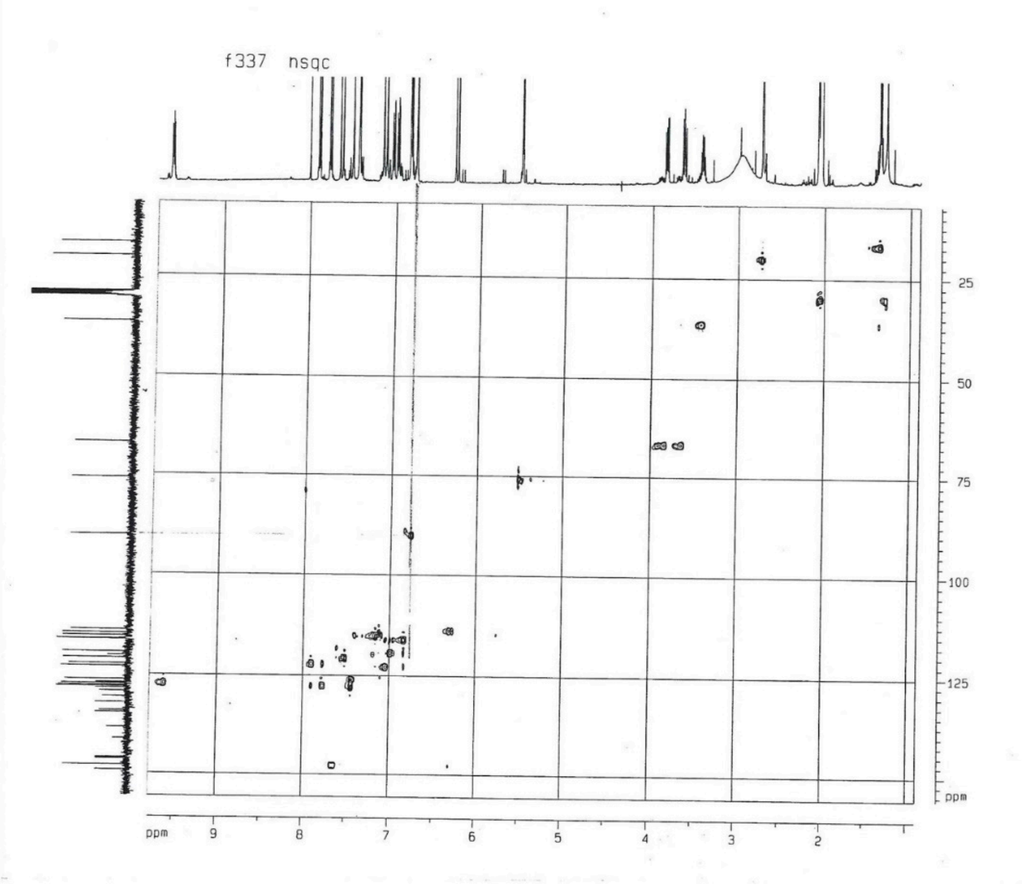

Figure S6. HSQC spectrum of **1** in acetone-d_6.

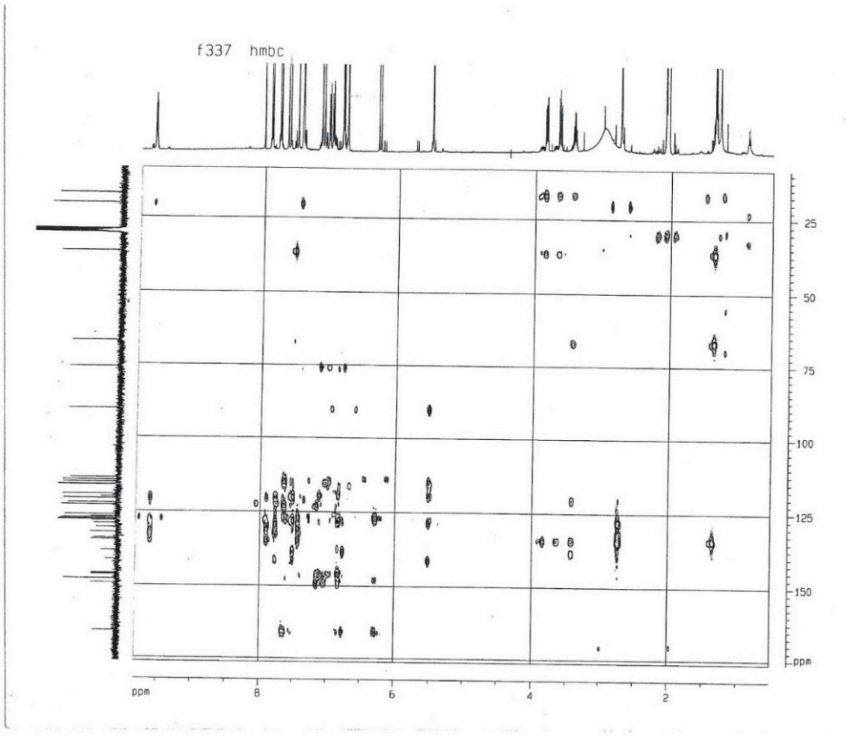

Figure S7. HMBC spectrum of **1** in acetone-*d*6.

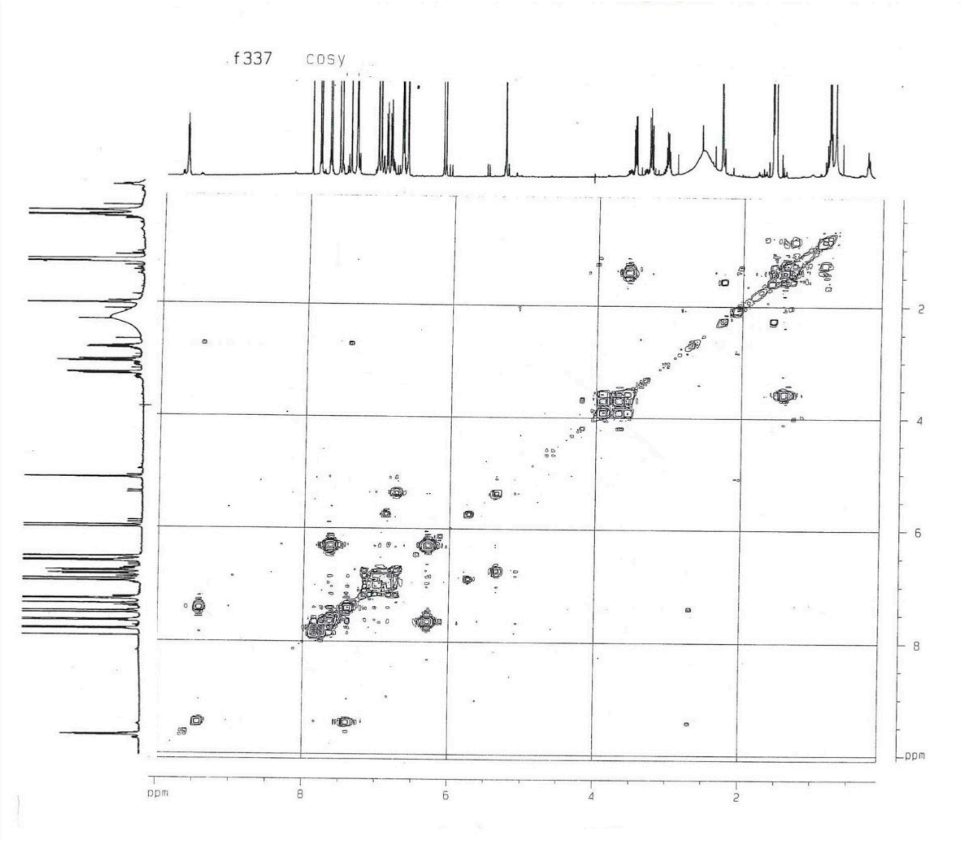

Figure S8. COSY spectrum of **1** in acetone-*d*6.

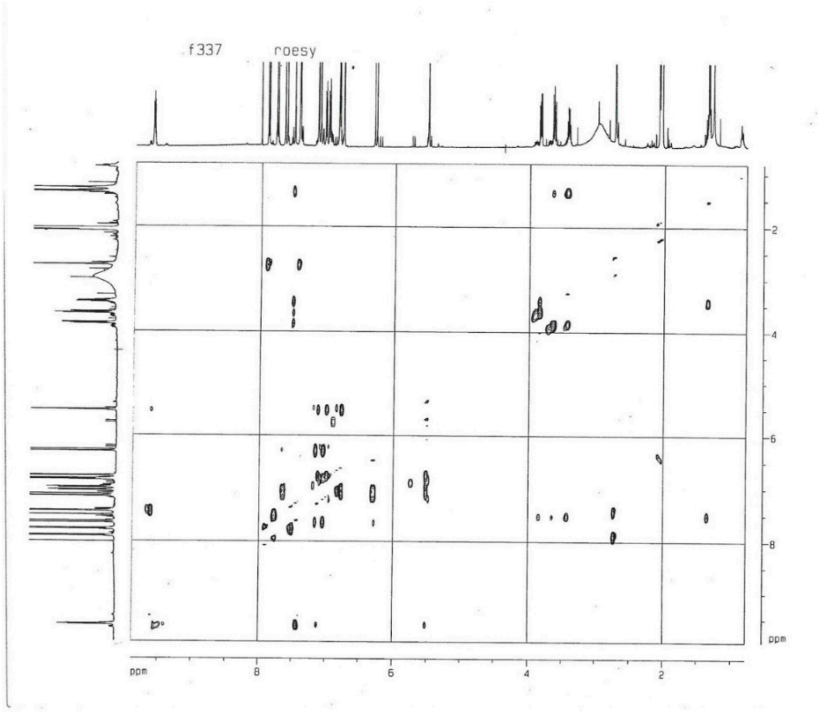

Figure S9. ROESY spectrum of **1** in acetone-*d*6.

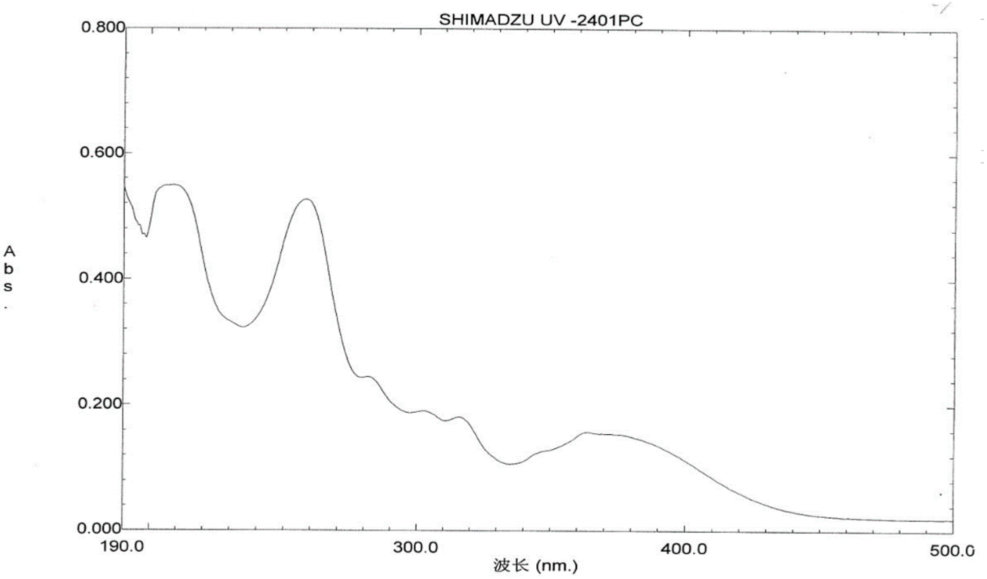

Figure S10. UV spectrum of **2** (MeOH).

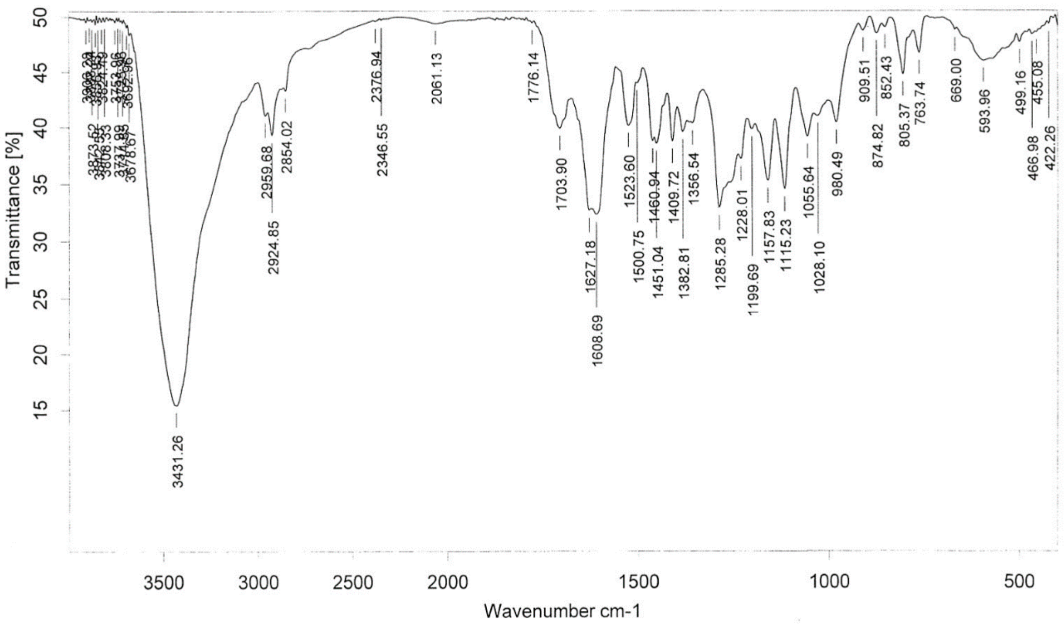

Figure S11. IR spectrum of **2** (KBr).

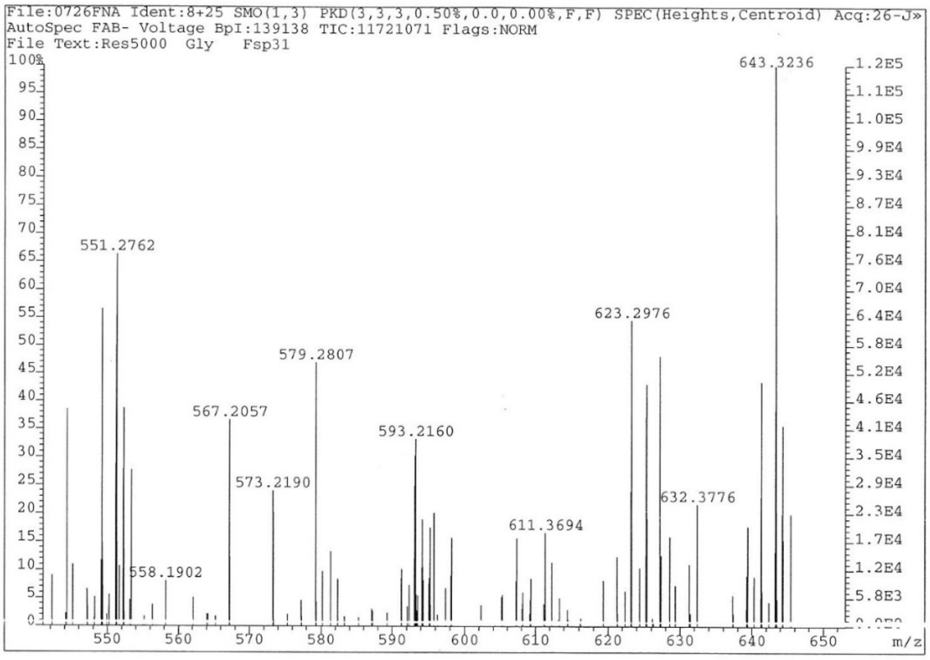

Figure S12. HRFABMS spectrum of **2** (KBr).

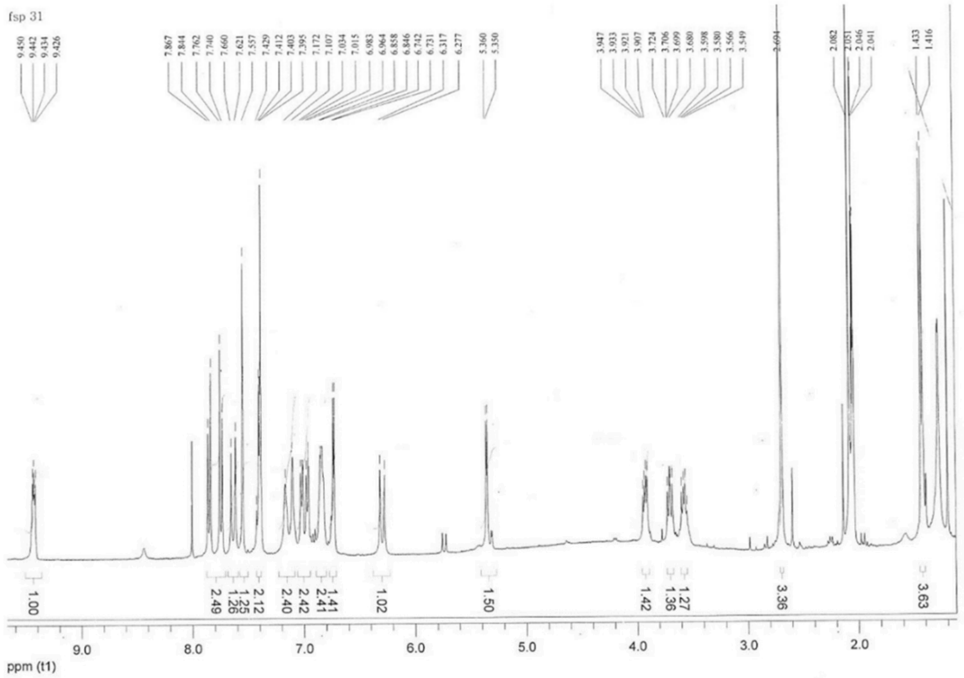

Figure S13. ^{1}H NMR spectrum of **2** in acetone-d_6.

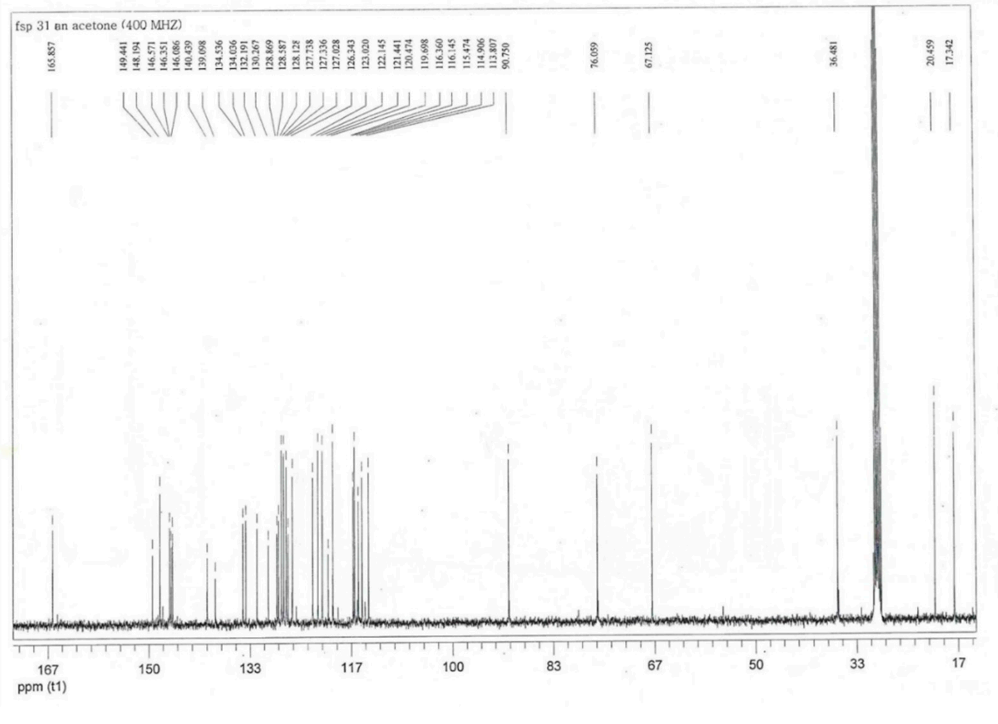

Figure S14. ^{13}C NMR spectrum of **2** in acetone-d_6.

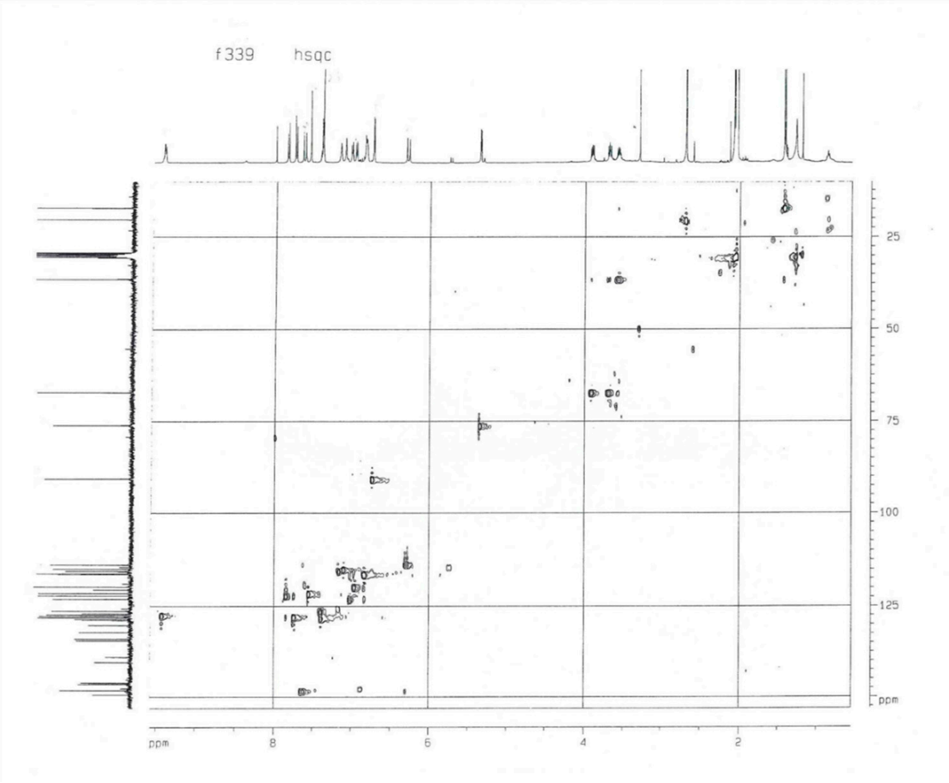

Figure S15. HSQC spectrum of **2** in acetone-*d*6.

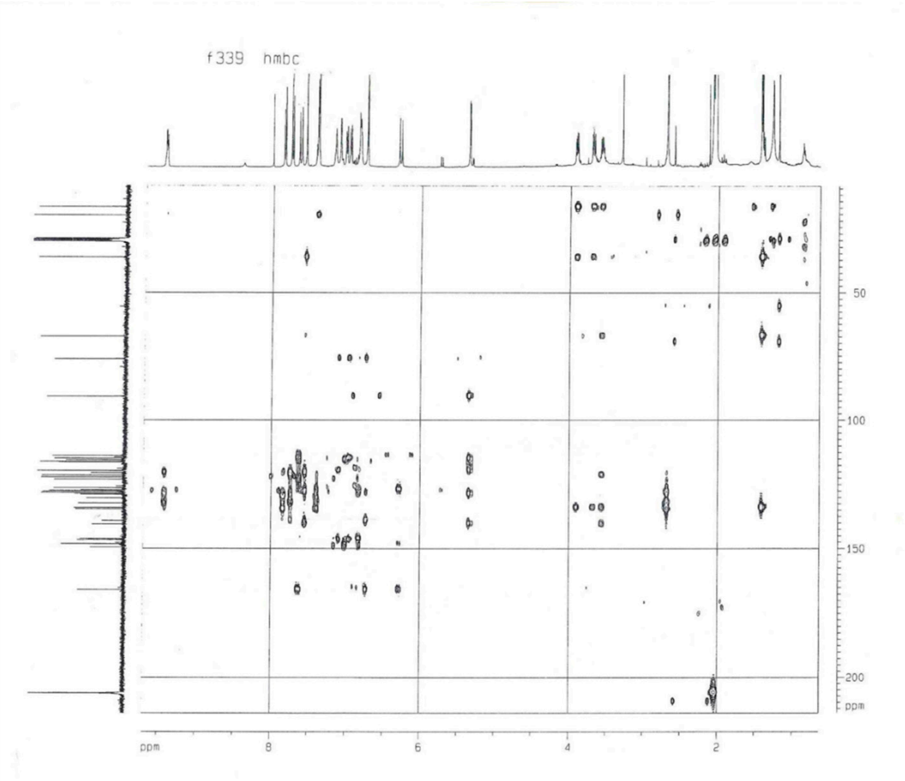

Figure S16. HMBC spectrum of **2** in acetone-*d*6.

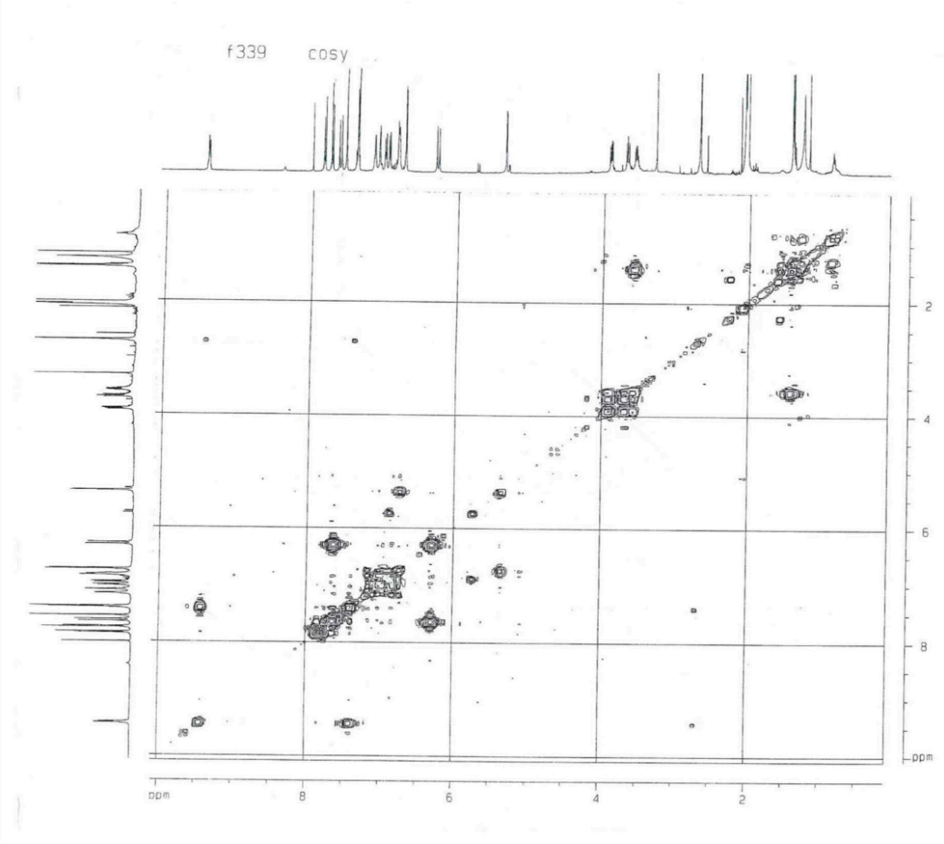

Figure S17. COSY spectrum of **2** in acetone-d_6.

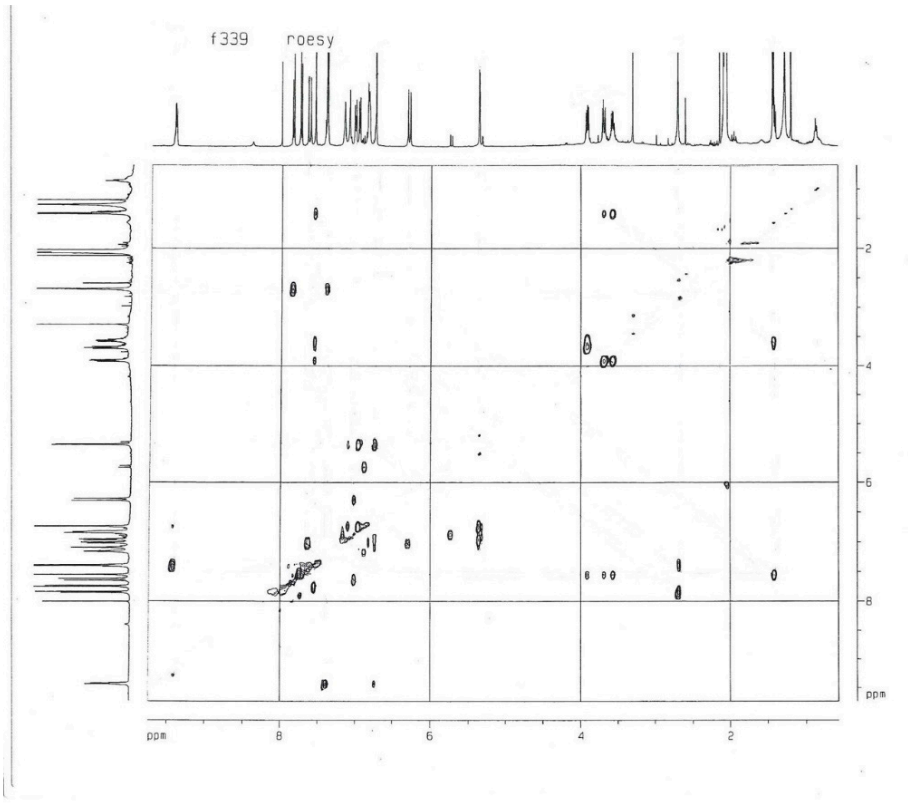

Figure S18. ROESY spectrum of **2** in acetone-d_6.
